# Concurrent mutations of germline *GPR101* and somatic *USP8* in a pediatric giant pituitary ACTH adenoma: a case report

**DOI:** 10.1186/s12902-022-01058-8

**Published:** 2022-06-06

**Authors:** Xu-dong Bao, Lin Lu, Hui-juan Zhu, Yong Yao, Ming Feng, Ren-zhi Wang, Xiao Zhai, Yong Fu, Feng-ying Gong, Zhao-lin Lu

**Affiliations:** 1 Department of Pediatrics, Peking Union Medical College Hospital, Chinese Academy of Medical Science and Peking Union Medical College, Beijing, 100730 China; 2 Department of Endocrinology, Key Laboratory of Endocrinology of National Health Commission, Peking Union Medical College Hospital, Chinese Academy of Medical Science and Peking Union Medical College, Beijing, 100730 China; 3 Department of Neurosurgery, Peking Union Medical College Hospital, Chinese Academy of Medical Science and Peking Union Medical College, Beijing, 100730 China

**Keywords:** *GPR101*, *USP8*, Cushing’s disease, Invasive, Pediatric

## Abstract

**Background:**

Cushing’s disease (CD) is rare in pediatric patients. It is characterized by elevated plasma adrenocorticotropic hormone (ACTH) from pituitary adenomas, with damage to multiple systems and development. In recent years, genetic studies have shed light on the etiology and several mutations have been identified in patients with CD.

**Case presentation:**

A girl presented at the age of 10 years and 9 months with facial plethora, hirsutism and acne. Her vision and eye movements were impaired. A quick weight gain and slow growth were also observed. Physical examination revealed central obesity, moon face, buffalo hump, supra-clavicular fat pads and bruising. Her plasma ACTH level ranged between 118 and 151 pg/ml, and sella enhanced MRI showed a giant pituitary tumor of 51.8 × 29.3 × 14.0 mm. Transsphenoidal pituitary debulk adenomectomy was performed and immunohistochemical staining confirmed an ACTH-secreting adenoma. Genetic analysis identified a novel germline *GPR101* (p.G169R) and a somatic *USP8* (p. S719del) mutation. They were hypothesized to impact tumor growth and function, respectively.

**Conclusions:**

We reported a rare case of pediatric giant pituitary ACTH adenoma and pointed out that unusual concurrent mutations might contribute to its early onset and large volume.

## Background

Cushing’s disease (CD) is caused by the overproduction of adrenocorticotropic hormone (ATCH) by pituitary adenomas (PAs). It is rare in children and accounts for approximately 75% of pediatric Cushing’s syndrome from 7 to 17 years of age [1]. Weight gain and facial changes are more common in children than in adults [2]. Growth retardation is also a characteristic of children with hypercortisolemia [3]. Genetic alterations such as somatic *USP8*, *RASD1*, *TP53* mutations, and germline *AIP*, *MEN1*, and *CABLES1* mutations have been identified in CD patients [4]. Here we report a case of pediatric invasive pituitary ACTH macroadenoma associated with a novel germline *GPR101* (p. G169R) and a somatic *USP8* (p. S719del) mutation.

## Case presentation

The girl was born at full term with a length of 48 cm and a weight of 2900 g. Her neuromotor and cognitive development was comparable to those of children of the same age. At the age of 9 years and 4 months she developed plethora, hirsutism, facial acne, rapid weight gain, and increased abdominal circumference. Her skin darkened, and purple striae appeared on thighs and in the armpits. She became dull and less talkative, as indicated by her parents. At 10 years and 3 months, the patient complained of pain around the left orbit with an intensity of 4–5 points on a numerical rating scale (NRS). Five months later bilateral blepharoptosis appeared, with significantly impaired vision of the left eye. Soon both eyes failed to rotate in all directions.

On admission the patient was 10 years and 9 months, with a height of 144 cm (90–97th percentile) and a weight of 48 kg (25–50th percentile). Her weight gain was 20 kg, while the height increased by only 2–3 cm in 18 months. Her blood pressure was 115/76mmHg, and her heart rate was 80 bpm. Apart from the signs mentioned above, physical examination revealed central obesity (BMI 23.1 kg/m^2^), moon face, buffalo hump, supra-clavicular fat pads and bruising at the left fossa cubitalis. Her pupils were 7 mm in diameter and barely reacted to light. There was a fan-shaped visual field defect in the left eye. Her breasts were Tanner stage III and pubic hair was Tanner stage II, although menarche had not yet occurred. The parents and her younger brother at 6 years of age did not have symptoms related to Cushing syndrome, acromegaly or gigantism. There was no family history of pituitary tumor or other endocrine tumors.

**Fig. 1:**
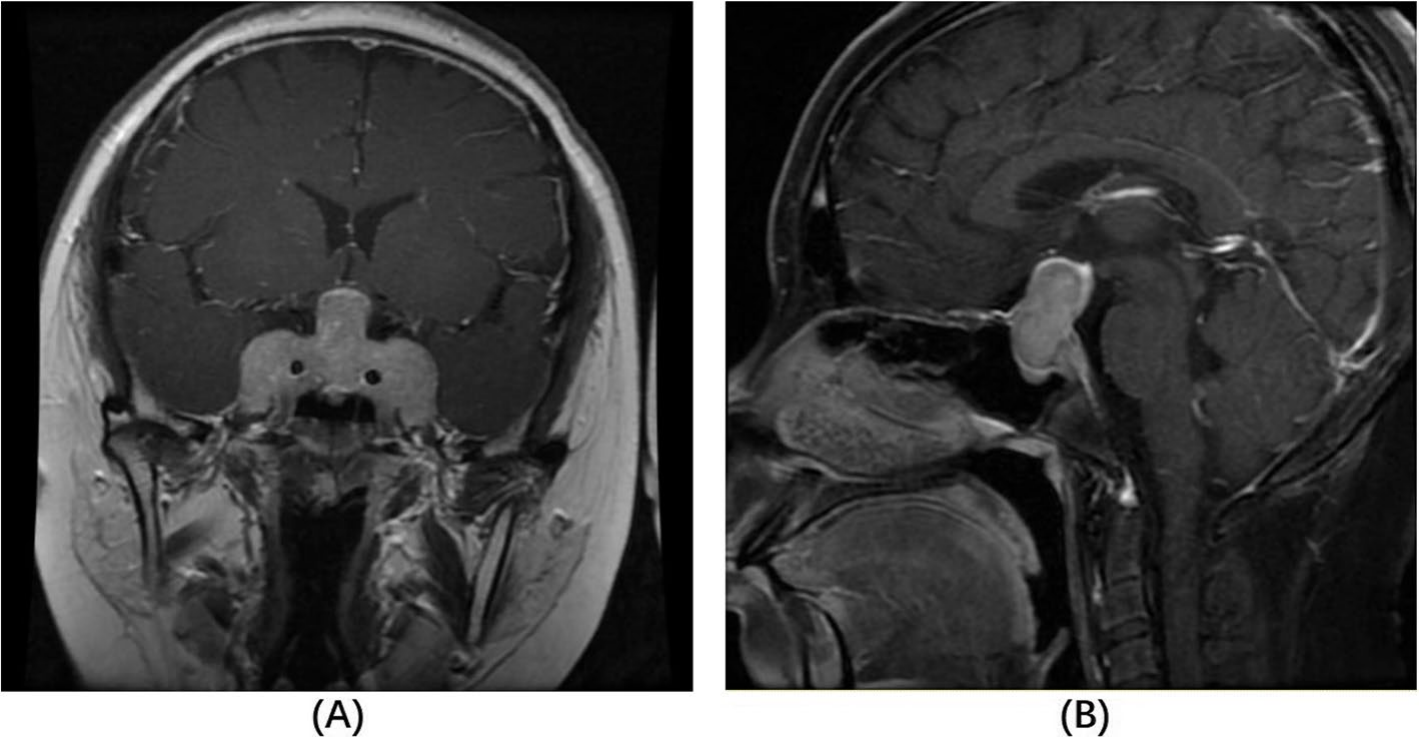
Contrast-enhanced coronal (**A**) and sagittal (**B**) T1-weighted MRI on admission. The sellar mass measured 51.8 × 29.3 × 14.0 cm (TD × VD × APD) with a heterogeneous density in the enhanced scan. The diaphragma sellea was dramatically elevated, with optic chiasm compressed. The sellar floor was sunken and bilateral cavernous sinus was surrounded (Knosp 4)

She had increased midnight serum cortisol (24.35 µg/dL, normal range < 1.8 µg/mL) and 24-hour urine free cortisol (24hUFC) (308.0 µg, normal range 12.3–103.5). The plasma ACTH level ranged from 118 to 151 pg/mL (< 46pg/mL). The 24hUFC was not suppressed (79.2 µg) after 48 h low-dose dexamethasone suppression test (LDDST), but suppressed to 32.8 µg (suppression rate 89.4%) after 48 h high-dose dexamethasone. Sella enhanced MRI showed a giant pituitary tumor measured 51.8 × 29.3 × 14.0 mm with heterogeneous density (Fig. 1). The mass compressed the optic chiasma and surrounded the bilateral cavernous sinus (Knosp 4). Therefore, an invasive giant pituitary ACTH adenoma was clinically diagnosed. The morning growth hormone (GH) was 1.0ng/ml (< 2 ng/ml) and insulin-like growth factor 1 416 ng/ml (88–452 ng/ml). The prolactin (PRL), luteinizing hormone (LH), follicle-stimulating hormone (FSH) and thyroid stimulating hormone (TSH) were all in normal ranges, as well as serum sodium, potassium, blood glucose and urine osmolality. Abdominal ultrasonography revealed a fatty liver. Tests concerning type 1 multiple endocrine neoplasia included serum calcium, phosphate, parathyroid hormone, gastrin and glucagon, which were all unremarkable (Table 1).

**Table 1:** Laboratory data on admission

Variable	Value	Reference range
GH (ng/ml)	1.0	< 2.0
IGF-1 (ng/ml)	416	88–452
TSH (µIU/mL)	1.937	0.380–4.340
FT3 (pg/ml)	1.19	1.80–4.10
FT4 (ng/dl)	0.77	0.81–1.89
FSH (IU/L)	0.31	0.8–4.6
LH (IU/L)	0.34	0.5–4.3
PRL (ng/ml)	7.0	< 30
Estradiol (pg/ml)	19	7–60
Testosterone (ng/ml)	0.57	< 0.6
DHEAs (µg/dl)	428	< 184
Morning ACTH (pg/ml)	108–151	< 46
24hUFC baseline (µg)	308	12.3-103.5
24hUFC after LDDST (µg)	79.2	/
24hUFC after HDDST (µg)	32.8	/
Gastrin (pg/ml)	17	13–115
Glucagon (pg/ml)	153.56	0-200
PTH (pg/mL)	36.6	12–68
Plasma Metanephrine (nmol/l)	0.07	< 0.9
Plasma Normetanephrine (nmol/l)	0.09	< 0.5
K (mmol/L)	4.6	3.5–5.5
Na (mmol/L)	140	135–145
Ca (mmol/L)	2.48	2.13–2.7
P (mmol/L)	1.34	0.81–1.45
TG (mmol/L)	0.48	0.45–1.7
Total cholesterol (mmol/L)	4.44	2.85–5.7
LDL-C (mmol/L)	2.29	< 3.37
HDL-C (mmol/L)	1.99	0.93–1.81
FPG (mmol/L)	4.8	3.9–6.1
HbA1c (%)	5.9	4.5–6.3
Fasting insulin (µIU/ml)	29.8	5.2–17.2

*GH *growth hormone, *IGF-1 *insulin-like growth factor 1, *TSH *thyroid stimulating hormone, *FT3 *free triiodothyronine, *FT4 *free thyroxine, *FSH *follicle-stimulating hormone, *LH *luteinizing hormone, *PRL *prolactin, *DHEAs *dehydroepiandrosterones, *PTH *parathyroid hormone, *K *serum potassium, *Na *serum sodium, *Ca *serum calcium, *P *serum phosphate, *TG *total glyceride, *LDL-C *low-density lipoprotein cholesterol, *HDL-C *high-density lipoprotein cholesterol, *FPG *fasting plasma glucose, *HbA1c *hemoglobin A1c

**Fig. 2:**
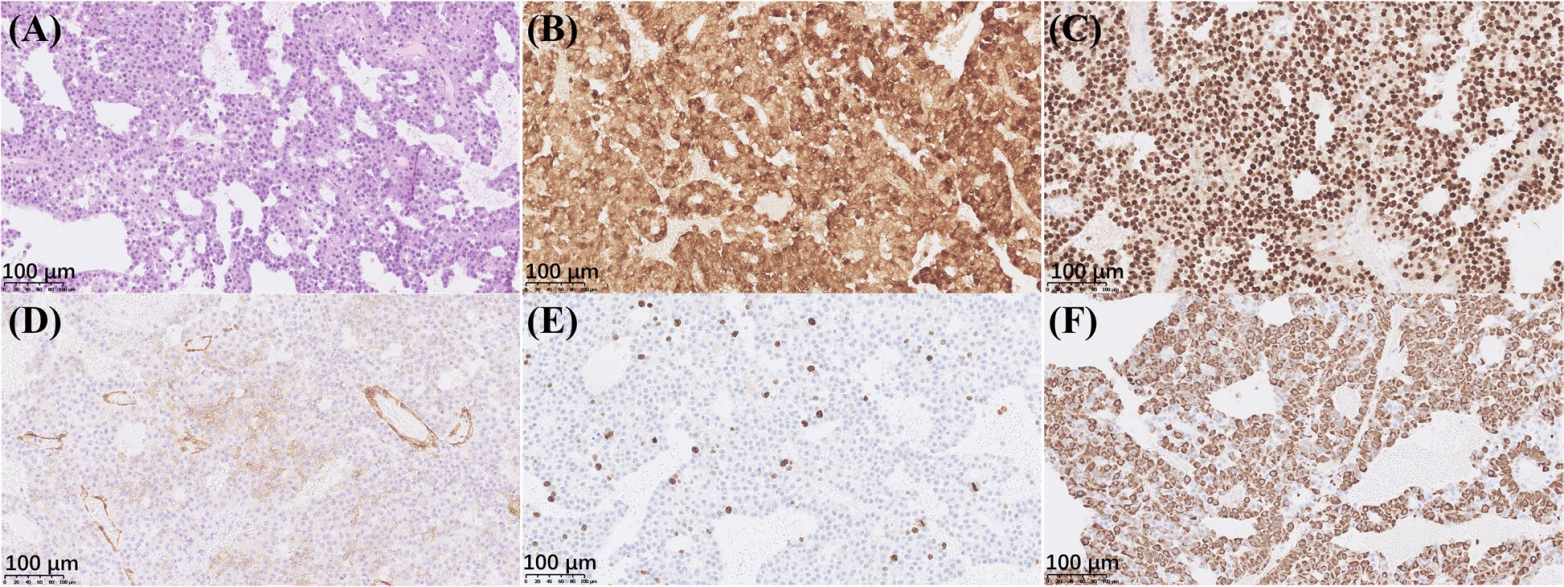
Histopathology and immunohistochemistry staining results of the pituitary tumor. By light microscopy, the tumor cells were mostly basophilic and arranged in papillary architecture. Neither necrosis nor mitotic activity was observed (**A** hematoxylin-eosin, ×200). Immunohistochemistry staining was positive for ACTH (**B** immunoperoxidase, ×200) and transcription factor T-PIT (**C** immunoperoxidase, ×200). Cytoplasmic staining of SSTR2A was observed in around 1/3 tumor cells besides the strong staining of endothelial cells (**D** immunoperoxidase, ×200). The Ki-67 index was 10% (E immunoperoxidase, ×200). Cytokeratin CAM5.2 was diffusely positive in the cytoplasm (**F** immunoperoxidase, ×200). The positive control for ACTH and T-PIT was the human anterior pituitary gland, and for SSRT2, Ki-67 and CAM5.2 were cerebral cortex, tonsil and colonic mucosa, respectively

Transsphenoidal pituitary debulk adenomectomy was performed immediately due to multiple cranial nerve involvement and the negative results of Sandostatin loading test. A decompression resection was done. The plasma ACTH level declined to 77 pg/ml and serum cortisol 30.2 µg/dl three days after the operation. Vision, pupil dilation, eye movements and blepharoptosis also partially improved. Histopathology and immunohistochemical staining confirmed a densely–granulated corticotroph adenoma (Fig. 2, NanoZoomer S360 digital slide scanner and NDP.view 2.9.25 software, Hamamatsu, Japan). Neither necrosis nor mitotic activity was observed. The immunostaining for somatostatin receptor SSTR2A was positive with a cytoplasmic pattern, while GH, PRL, TSH, FSH, LH and PIT were all negative. The Ki 67 index was found to be 10%. One month after the operation the ACTH level increased to 132 pg/mL again, and the parents agreed to refer their child for radiotherapy to control the residual tumor.

The early onset and invasive behavior of this tumor led to the consideration of whether there was a genetic defect. Genetic studies were recommended for the families and they all agreed and signed the written informed consent forms. Whole exome sequencing (WES) was performed on the patient’s blood sample using an Illumina HiSeq sequencer to an average read depth of at least 90 times per individual. Raw sequence files were mapped to the GRCH37 human reference genome and analyzed using the Sentieon software. The results revealed a germline heterozygous *GPR101* gene mutation c.505G > C (p.Gly169Arg), which was subsequently confirmed to be of maternal origin by Sanger sequencing. Meanwhile WES of the tumor tissue identified an additional somatic heterozygous c.2155_2157delTCC (p.S719del) mutation of the *USP8* gene .

## Discussion and conclusions

In this report, we described an extremely giant and invasive pituitary ACTH adenoma in a 10-year-old girl. According to Trouillas et al., invasive and proliferative pituitary tumors have a poor prognosis [5]. CD is rare among children, and the fast-growing and invasive nature of the tumor in this case led to the investigation of genetic causes. The somatic *USP8* gene mutation has been recently reported to be associated with the pathogenesis of CD [6, 7]. This gene encodes ubiquitin-specific protease 8 (USP8). S718, S719 and P720 are hotspots in different studies [6–14]. They are located at the 14-3-3 binding motif, and the mutations disrupt the binding between USP8 and 14-3-3 protein, which leads to increased deubiquitination and EGFR signaling. High levels of EGFR consequently trigger proopiomelanocortin (POMC) transcription and ACTH secretion [6, 7]. The p.S719del mutation has been previously reported and its pathogenicity has been confirmed [7]. Thus, we speculate the p.S719del mutation plays a role in this patient with CD.

It is noteworthy that in our case, the pituitary corticotrophin adenoma was extremely giant and bilaterally invasive. *USP8* mutations have been found in 31% of pediatric CD patients [10]. It is well known that microadenomas are most common in adult and pediatric CD patients. Previously, the Chinese and Japanese cohorts observed smaller sizes of *USP8*-mutated PAs than wild-type PAs [7, 9]. The Chinese cohort also reported a lower rate of invasive adenomas in *USP8*-mutated PAs [7]. This may be explained by the finding that *UPS8* mutations did not significantly promote cell proliferation more than the wild-type ones [6]. Other cohorts suggested no difference in tumor size or invasiveness between *USP8*-mutated and wild-type PAs [8, 10, 12–14], which may be partially explained by the differences in sample sizes and ethnic backgrounds. Owing to the lack of evidence of *USP8* mutations significantly contributing to tumor growth and invasiveness, additional pathogenesis should be investigated in this case.

The p.Gly169Arg mutation of the *GPR101* gene has not been reported in patients with pituitary tumors. *In silico* predictions were performed using Polyphen-2, Mutation Taster and PROVEAN, and all of the programs reported it to be pathogenic. The *GPR101* gene encodes an orphan G protein-coupled receptor (GPCR) and microduplication encompassing the gene has been proven to be the cause of X-linked acrogigantism (XLAG) [15]. XLAG is characterized by the early onset of pituitary GH-secreting macroadenomas. Point mutations of *GPR101* have been found in patients with PAs that are mostly GH-secreting [15–17]. Although their prevalence is very low, an *in vitro* study supported the pathogenic role of p.E308D, the most common mutation of *GPR101*. This led to increased cell proliferation and GH production in rat pituitary GH3 cells [15]. Rare cases of PRL, ACTH or TSH-secreting PAs with *GPR101* variants were also documented [16, 18]. To date, there have been five cases of ACTH-secreting PAs with four different germline *GPR101* mutations: two cases of p.E308D, p.I122T, p.T293I and p.G31S, although *in silico* predictions and *in vitro* evaluations using AtT-20 cells have respectively determined the latter two mutations to be non-pathogenic [16, 18]. These patients were mainly children and young adults. Unlike pituitary GH-secreting tumors, the role of *GPR101* mutations in the pathophysiology of CD is still questionable. Trivellin et al. demonstrated no statistically significant difference in *GPR101* expression between corticotropinomas and normal human pituitaries. No significant correlation between *GPR101* and POMC expression levels was found neither [18].

Given the evidences above, we hypothesize that the somatic *USP8* mutation is responsible for the overexpression of ACTH in this CD girl while the germline *GPR101* mutation contributes to the early onset and fast-growing nature of the tumor. Similarly, a 27-year-old woman with Nelson’s syndrome originally considered to be associated with a germline *AIP* variant (p.Arg304Gln) was recently reported to have a somatic *USP8* mutation. The patient progressed rapidly and underwent multiple transsphenoidal surgeries [19]. Since germline *AIP* mutations are more commonly seen in GH-secreting PAs [20], the authors proposed that the *USP8* mutation might have shifted the tumor towards ACTH-secreting [19]. Further investigations into the pathogenicity of *GPR101* p.Gly169Arg and *AIP* p.Arg304Gln mutations are required to support the hypothesis.

In summary, we report a novel germline *GPR101* and somatic *USP8* mutation in a girl with an extremely giant pituitary ACTH adenoma. The concurrent mutations may lead to the growth and function of the tumor, respectively. Further investigations should be carried out to verify the role of the concurrent mutations in the pathogenesis of pediatric CD.

## Data Availability

The WES data of the blood sample of the patient is available in the NGDC repository (https://ngdc.cncb.ac.cn/gsa-human/) and the accession number is HRA002396. Any additional information is available from the authors upon reasonable request.

## Abbreviations

CD: Cushing’s disease
ACTH: adrenocorticotropic hormone
PA: pituitary adenoma
NRS: numerical rating scale
24hUFC: 24-hour urine free cortisol
LDDST: low-dose dexamethasone suppression test
USP8: ubiquitin-specific protease 8
POMC: proopiomelanocortin
GPCR: G protein-coupled receptor
XLAG: X-linked acrogigantism
